# Rituximab maintenance overcomes the negative prognostic factor of obesity in CLL: Subgroup analysis of the international randomized AGMT CLL‐8a mabtenance trial

**DOI:** 10.1002/cam4.1980

**Published:** 2019-03-19

**Authors:** Alexander Egle, Thomas Melchardt, Petra Obrtlíková, Lukáš Smolej, Tomáš Kozák, Michael Steurer, Johannes Andel, Sonja Burgstaller, Eva Mikušková, Liana Gercheva, Thomas Nösslinger, Tomáš Papajík, Miriam Ladická, Michael Girschikofsky, Mikuláš Hrubiško, Ulrich Jäger, Daniela Voskova, Martin Pecherstorfer, Eva Králiková, Christina Burcoveanu, Emil Spasov, Andreas Petzer, Georgi Mihaylov, Julian Raynov, Horst Oexle, August Zabernigg, Emília Flochová, Stanislav Palášthy, Olga Stehlíková, Michael Doubek, Petra Altenhofer, Lukas Weiss, Teresa Magnes, Lisa Pleyer, Anton Klingler, Jiří Mayer, Richard Greil

**Affiliations:** ^1^ Third Medical Department Paracelsus Medical University Salzburg Salzburg Austria; ^2^ Salzburg Cancer Research Institute (SCRI) Salzburg Austria; ^3^ Cancer Cluster Salzburg (CCS) Salzburg Austria; ^4^ First Faculty of Medicine Charles University in Prague and General University Hospital in Prague Prague Czech Republic; ^5^ Fourth Department of Internal Medicine ‐ Hematology, Faculty of Medicine in Hradec Králové University Hospital and Charles University in Prague Hradec Králové Czech Republic; ^6^ Department of Internal Medicine ‐ Hematology University Hospital Kralovske Vinohrady Prague Czech Republic; ^7^ Department of Internal Medicine V Medical University Innsbruck Innsbruck Austria; ^8^ Internal Medicine II Hospital of Steyr Steyr Austria; ^9^ Department of Internal Medicine IV Klinikum Wels‐Grieskirchen GmbH Wels Austria; ^10^ Department of Hemato‐oncology 2 National Cancer Institute Bratislava Bratislava Slovakia; ^11^ Clinic of Hematology University Hospital St Marina Varna Varna Bulgaria; ^12^ Third Medical Department for Hematology and Oncology Hanusch Krankenhaus der Wiener Gebietskrankenkasse Vienna Austria; ^13^ Department of Hemato‐oncology University Hospital Olomouc Olomouc Czech Republic; ^14^ Department of Clinical Oncology 1 National Cancer Institute Bratislava Bratislava Slovakia; ^15^ Interne Abteilung Ordensklinikum Linz GmbH Elisabethinen, Linz Austria; ^16^ Clinic of Hematology and Transfusiology Slovak Medical University, University Hospital Bratislava Bratislava Slovakia; ^17^ Department of Medicine I, Division of Hematology and Hemostaeology Medical University Vienna Vienna Austria; ^18^ Department of Internal Medicine 3 Kepler Universitätsklinikum GmbH, Med Campus III. Linz Austria; ^19^ Department of Internal Medicine 2 University Hospital Krems, Karl Landsteiner Private University of Health Sciences Krems Austria; ^20^ Department of Hematology, FNsP F D Roosevelta Banská Bystrica Banska Bystrica Slovakia; ^21^ Clinic of Haematology Regional Institute of Oncology Iasi Iasi Romania; ^22^ Clinic of Hematology UMHAT St George and Medical University Plovdiv Plovdiv Bulgaria; ^23^ Innere Medizin I, Ordensklinikum Linz GmbH Linz Austria; ^24^ Hematological Clinic NSHATHD Sofia, Queen Joanna University Hospital Sofia Bulgaria; ^25^ Clinic of Medical Hematology Military Medical Academy Sofia Sofia Bulgaria; ^26^ Innere Medizin Landeskrankenhaus Hall Hall in Tirol Austria; ^27^ Innere Medizin II Bezirkskrankenhaus Kufstein Kufstein Austria; ^28^ Department of Hematology and Transfusion University Hospital Martin Martin Slovakia; ^29^ Department of Clinical Hematology FNsP, J A Reimana Prešov Prešov Slovakia; ^30^ Faculty of Medicine and CEITEC University Hospital Brno Brno Czech Republic; ^31^ Assign Data Management and Biostatistics GmbH Innsbruck Austria; ^32^ Present address: Reha Zentrum Münster Münster Austria

**Keywords:** BMI, CLL, maintenance, obesity, rituximab

## Abstract

No data are available regarding obesity and outcome in Chronic Lymphocytic Leukemia (CLL). We analyzed 263 patients from the AGMT CLL‐8a Mabtenance trial for the impact of obesity. The trial included patients after rituximab‐containing induction treatment in first or second line that had achieved at least a PR. A randomization to rituximab maintenance treatment (375 mg/m^2^ q3 months for 2 years) vs observation was performed. In this cohort 22% of the patients (58/263) were classified as obese. The baseline response to induction treatment was inferior in obese patients with a lower CR rate (43.1% vs 60.5% in obese vs non‐obese, *P* = 0.018) and with a lower rate of patients achieving MRD negativity after chemoimmunotherapy induction treatment (19.6% vs 35.8%, *P* = 0.02). The PFS outcome of obese patients was significantly worse in the observation group of the trial (24 vs 39 months median PFS, *P* = 0.03). However, in the rituximab maintenance group the outcome for obese vs non‐obese was not different (*P* = 0.4). In summary, obesity was overall associated with a worse outcome of chemoimmunotherapy induction. However, rituximab maintenance treatment seems to be able to overcome this negative effect.

## Graphical abstract

We analyzed 263 patients from the AGMT CLL‐8a Mabtenance trial for the impact of obesity. The trial included patients after rituximab‐containing induction treatment in first or second line that had achieved at least a PR. Obesity was overall associated with a worse outcome of chemoimmunotherapy induction.

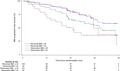

To the Editor:

Obesity has been identified as a risk factor for the development of solid tumors and lymphoid malignancies. A body mass index higher than 30 kg/m^2^ has been estimated to cause 20% of all cancers worldwide. Obese patients also have a higher incidence of non‐Hodgkin lymphomas than patients with normal weight. In addition, obesity is linked to inferior outcomes in established cancer diagnoses, but this excess mortality may be caused in part by higher rates of cardiovascular comorbidities.^1^

There are conflicting results regarding the prognostic role of higher BMI during treatment for aggressive lymphoma and indolent lymphoma. A negative effect of higher body weight is thought to be partly explained by higher rituximab clearance, with an additional role of sex.^2^, ^3^ Obesity has also been investigated as a risk factor for the development of chronic lymphocytic leukemia (CLL), with conflicting results to date. Nevertheless, the prognostic role of obesity during modern treatment of CLL has not yet been explored. Therefore, we performed an exploratory analysis to define the role of obesity in patients treated in the prospective phase III CLL‐8 trial.

After a rituximab‐containing induction treatment, 263 patients were randomized to either standard observation after treatment or rituximab maintenance for 2 years, as previously described. At a median observation time of 33.4 months, the HR (hazard ratio) for PFS as the primary endpoint of the trial was significantly in favor of rituximab maintenance (HR: 0.5, *P *= 0.0007), as previously reported.^4^

The median BMI for the entire cohort was 26.9, ranging from 17.1 to 40.1. There was no difference regarding the median BMI between the two treatment groups (26.8 and 27.1, respectively). Obesity, defined as a BMI above 30, was diagnosed at study entry in 22.1% of all patients. There were no differences with respect to line of therapy, median age or distribution of gender in obese and non‐obese patients; however, there was a trend toward more high‐risk cytogenetics (del11q or del17p) in non‐obese patients (35.1% vs 20%, *P *= 0.05; for details, see Table 1A). Despite this fact, obese patients had a lower rate of CR to the last chemoimmunotherapy (43.1% vs 60.0%, *P *= 0.018) and of MRD negativity in the bone marrow (19.6% vs 35.8%, *P *= 0.02) at study entry compared with non‐obese patients in either cohort (Table 1B).

**Table 1:** Patient characteristics and remission status of patients treated with chemoimmunotherapy (A) and Cox regression analysis of prognostic factors (B)

A	Overall (n = 263)	non‐obese (n = 205)	Obese (n = 58)	*P*‐value
Mean age, years (range, ± SD)	62.9 (35‐85, 9.5)	62.9 (35‐85, 9.7)	62.9 (41‐79, 8.8)	*0.97* A
Sex				
Male (%)	71.1	70.7	72.4	*0.80* B
Female (%)	28.9	29.3	27.6	
Line of treatment				
First‐line treatment (%)	79.8	81.5	74.1	*0.21* B
Second‐line treatment (%)	20.2	18.5	25.9	
Last induction treatment				
FCR (%)	73.4	73.7	72.4	*0.85* B
Others (%)	26.6	26.3	27.6	
Cytogenetic: del11q or del17p				
Yes (%)	31.9	35.1	20.0	*0.05* B
No (%)	68.1	64.9	80.0	
*Available (*n*/total)*	*213/263*	*168/205*	*45/58*	
MRD Peripheral blood				
Positive (%)	44.2	42	51.9	*0.20* B
Negative (%)	55.8	58	48.1	
*Available (*n*/total)*	*242/263*	*188/205*	*54/58*	
MRD Bone marrow				
Positive (%)	44.2	64.2	80.4	*0.04* B
Negative (%)	55.8	35.8	19.6	0.02
*Available (*n*/total)*	*227/263*	*176/205*	*51/58*	
Response to last induction				
CR/CRi (%)	56.3	60	43.1	*0.018* B
PR (%)	43.7	40	56.9	

B	Univariate			Multivariate		
	HR	95% CI	*P*‐value	HR	95% CI	*P*‐value
Type of treatment						
First line vs second line	0.40	0.26‐0.62	*<0.001*	0.40	0.24‐0.66	*<0.001*
Response to last induction						
CR/CRi vs PR	0.44	0.30‐0.66	*<0.001*	0.45	0.28‐0.73	*0.001*
Treatment						
Maintenance vs observation	0.58	0.39‐0.87	*0.008*	0.46	0.28‐0.74	*0.002*
Cytogenetics						
del17p or del11q yes vs no	0.62	0.40‐0.95	*0.032*	0.66	0.41‐1.06	*0.09*
Peripheral blood						
MRD neg vs pos	0.24	0.16‐0.38	*<0.001*	0.54	0.32‐0.92	*0.024*
Bone marrow						
MRD neg vs pos	0.12	0.05‐0.25	*<0.001*	0.26	0.11‐0.61	*0.02*
Body mass index						
BMI<30 vs BMI>30	0.63	0.41‐0.97	*0.038*	0.57	0.35‐0.95	*0.033*

AMann‐Whitney‐U test, BPearson Chi‐Square.

BMI, body mass index; CI, confidence interval; CR, Complete remission; CRi, complete response with incomplete marrow recovery; FCR, fludarabin, cyclophosphamide and rituximab; HR, hazard ratio; MRD, minimal residual disease; PR, partial response; SD: standard deviation.

Italic values indicate the number of available/total patients included into the analysis.

Consequently, obese patients in the observational arm had inferior clinical outcomes compared to non‐obese patients (median PFS 23.6 vs 38.4 months, HR: 0.55, CI: 0.31‐0.97, *P *= 0.042; Figure 1).

**Figure 1:**
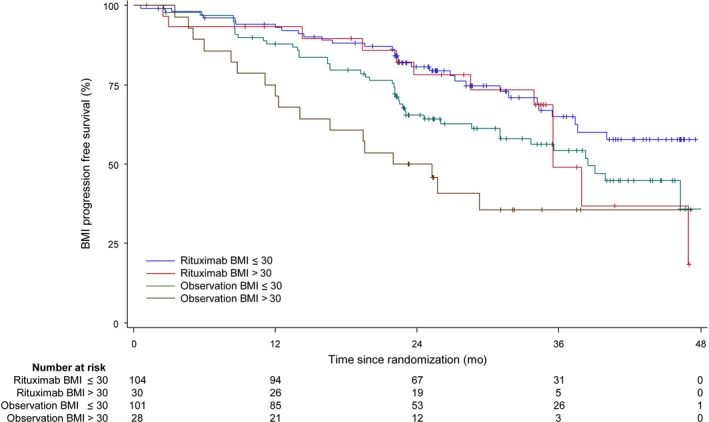
Progression‐free survival in the ITT population according to treatment and BMI. The median progression‐free survival was not influenced by obesity in patients treated with rituximab maintenance (median PFS 35.5 months vs not reached, HR: 0.75, CI: 0.38‐1.47, *P *= 0.41). In patients randomized to observation, obesity was associated with a worse median progression‐free survival (median PFS 23.6 vs 38.4 months, HR: 0.55, CI: 0.31‐0.97, *P *= 0.042)

Higher body weight is associated with a faster clearance of rituximab in patients with DLBCL.^3^ This may explain in part why male patients with DLBCL have an inferior prognosis with rituximab‐based polychemotherapy and may have the most benefit from more intensive rituximab regimens.^5^, ^6^ Rituximab is essential for achieving deep remissions in CLL, as shown in a randomized clinical trial where the incorporation of rituximab in the induction treatment increased the MRD negativity rate from 28% to 44%.^7^ Thus, inferior pharmacodynamics of rituximab in obese CLL patients may lead to inferior outcomes in obese patients treated with chemoimmunotherapy initially.

Considering the randomized design of our study, we were able to test whether rituximab maintenance treatment after chemoimmunotherapy was able to overcome this detrimental effect. When comparing the baseline characteristics between both arms and between obese *versus* non‐obese patients, we found no significant differences (data not shown). We did not observe a negative effect of obesity in patients randomized to rituximab maintenance strategy (median PFS 35.5 months vs not reached, HR: 0.75, CI: 0.38‐1.47, *P *= 0.41; Figure 1), which suggests that prolonged treatment may overcome the negative effect of obesity in the observational cohort. Consistent with this result, the benefit of rituximab maintenance was more clinically pronounced for the median PFS in obese patients (35.5 vs 23.6 months, HR: 0.46, CI: 0.22‐0.97, *P *= 0.041) compared to non‐obese patients (not reached vs 38.4 months, HR: 0.61, CI: 0.38‐0.96, *P *= 0.032), while remaining significant in both groups.

To assess the independent prognostic relevance of obesity as new risk factor it was added in a multivariate testing to risk factors, which had already been tested in the same data set of the Austrian CLL‐8 trial^4^ (see also Table 1B). This added obesity as a new independent prognostic factor (HR: 0.57; CI: 0.35‐0.95, *P *= 0.033) to the MRD status of peripheral blood (HR: 0.54; CI: 0.32‐0.92, *P *= 0.024) and bone marrow (HR: 0.26; CI: 0.11‐0.61, *P *= 0.002), response to induction treatment (HR: 0.45; CI: 0.28‐0.73, *P *= 0.001), line of induction treatment (HR: 0.40; CI: 0.24‐0.66, *P *< 0.001) and treatment group (HR: 0.46; CI: 0.28‐0.74, *P *= 0.002). As previously reported and discussed, cytogenetic status, including del17p and del11q, had no significant negative prognostic role in multivariate testing (*P *= 0.09).^4^

To the best of our knowledge, this is the first report showing the negative prognostic role of obesity in CLL patients treated with chemoimmunotherapy. While the role of obesity for the development of CLL remains unclear, we show that obese patients treated with state‐of‐the‐art treatment regimens incorporating rituximab achieved a lower rate CR and MRD negative remissions, which resulted in a lower PFS.

Different pharmacokinetics of rituximab in patients with a higher body mass index may play a role, as suggested by previous results in lymphoma patients with higher body mass indexes.^2^, ^3^ Other reasons for the inferior outcome in obese patients may be related to the lower levels of vitamin D found to be associated with obesity,^8^ or it may be a result of the decreased efficacy of antibody‐mediated cellular cytotoxicity. Regardless of mechanism, rituximab maintenance was able to overcome this negative effect of obesity and provide the same outcomes in obese and non‐obese CLL patients, which suggests that increased rituximab exposure may be especially important for obese populations. This finding may be very relevant in populations with increasing BMI, such as western societies, and it should be confirmed in other trials.

## CONFLICT OF INTEREST

SB receives support from Roche. AE receives grants and personal fees from Roche. RG receives grants and personal fees from Roche and Takeda, grants from Celgene and Novartis and personal fees from BMS and Amgen. MH receives personal fees from the Czech Lymphoma Study Group, and other support from Roche. TK receives other support from Roche. UJ receives grants and personal fees from Roche. AK receives personal fees from AGMT, Hoffmann‐La Roche, the Central European Society for Anticancer Drug Research and Arbeitsgemeinschaft Internistische Onkologie. JM receives other support from Roche, GlaxoSmithKline and Novartis. HO receives personal fees from Sanofi‐Aventis, Amgen and Celgene. PO receives grants from Roche, nonfinancial support from Janssen and Roche, and personal fees from GlaxoSmithKline and Gilead. AP receives personal fees from Roche. TP receives personal fees from Roche. LS receives personal fees from Abbvie, and personal fees and nonfinancial support from Gilead, Janssen and Roche. MS receives grants and personal fees from Roche. JA, PA, CB, MD, EF, LG, MG, EK, ML, EM, GM, Te.M., Th.M., TN, LP, MP, SP, JR, ES, OS, DV, LW and AZ declare no competing interests.
