# Supplementary material for: Rituximab maintenance overcomes the negative prognostic factor of obesity in CLL: Subgroup analysis of the international randomized AGMT CLL‐8a mabtenance trial
Source: Cancer Med. 2019 Mar 19;8(4):1401–5. doi: 10.1002/cam4.1980 (PMC6488104; doi:10.1002/cam4.1980)
Supplement: Supplementary file 1 [file CAM4-8-1401-s001.doc]

Supporting Information

for

**Rituximab maintenance overcomes the negative prognostic factor of obesity in CLL: Subgroup analysis of the international randomized AGMT CLL-8a mabtenance trial**

[Alexander Egle, MD](mailto:a.egle@salk.at)^1,2,3*^, Thomas Melchardt, MD^1,2,3*^, Petra Obrtlíková, MD^4^, Lukáš Smolej, MD^5^, Tomáš Kozák, MD^6^, Michael Steurer, MD^7^, Johannes Andel, MD^8^, Sonja Burgstaller, MD^9^, Eva Mikušková, MD^10^, Liana Gercheva, MD^11^, Thomas Nösslinger, MD^12^, Tomáš Papajík, MD^13^, Miriam Ladická, MD^14^, Michael Girschikofsky, MD^15^, Mikuláš Hrubiško, MD^16^, Ulrich Jäger, MD^17^, Daniela Voskova, MD^18^, Martin Pecherstorfer, MD^19^, Eva Králiková, MD^20^, Christina Burcoveanu, MD^21^, Emil Spasov, MD^22^, Andreas Petzer, MD^23^, Georgi Mihaylov, MD^24^, Julian Raynov, MD^25^, Horst Oexle, MD^26^, August Zabernigg, MD^27^, Emília Flochová, MD^28^, Stanislav Palášthy, MD^29^, Olga Stehlíková^30^, Michael Doubek, MD^30^, Petra Altenhofer, PhD^1,2,3^, Lukas Weiss, PhD^1,2,3^, Teresa Magnes, MD^1,2,3^, Lisa Pleyer, MD^1,2,3^, Anton Klingler, PhD^31^, Jiří Mayer, MD^30^ and Richard Greil, MD^1,2,3^

*This correspondence is in reference to Greil et al. [1]*

Methods

Patients eligible for this randomized, open-label, phase III trial had achieved a response after first or second line therapy rituximab-containing chemoimmunotherapy for B-CLL. Detailed inclusion and exclusion criteria were previously described.[2] After at least 4 cycles of chemoimmunotherapy patients were randomized to receive either rituximab 375 mg/m^2^ intravenously plus standard co-medication or to undergo observation at 3-monthly intervals, for a period of 2 years or until disease progression.

Minimal residual disease was defined according to the international standardized approach of the European Research Initiative on CLL using a 4 color-flow cytometry as already reported.[2]

Body Mass Index (BMI) was calculated in this post-hoc analysis according to the standard formula (BMI = weight (kg)/ height (m^2^)) at study entry after induction treatment and obesity was defined according to the standard WHO cutoff of BMI >30. Statistical analyses were performed using IBM® SPSS® statistics software, version 21. Mann-Whitney-U-test and Pearson’s Chi-Squared test were used for univariate analyses, where appropriate. Survival was estimated using Kaplan-Meier curve analysis, with statistical comparison using the log-rank test. A two-tailed significance-level of 0.05 was considered statistically significant. The BMI was added to the previously used multivariate model.

**References**

1. Greil, R., et al., *Rituximab maintenance versus observation alone in patients with chronic lymphocytic leukaemia who respond to first-line or second-line rituximab-containing chemoimmunotherapy: final results of the AGMT CLL-8a Mabtenance randomised trial.* Lancet Haematol, 2016. **3**(7): p. e317-29.

2. Greil, R., et al., *Rituximab maintenance versus observation alone in patients with chronic lymphocytic leukaemia who respond to first-line or second-line rituximab-containing chemoimmunotherapy: final results of the AGMT CLL-8a Mabtenance randomised trial.* Lancet Haematol., 2016. **3**(7): p. e317-e329.
